# Targeting Voltage-Gated Potassium Channels in Breast Cancer: Mechanistic Insights into 4-Aminopyridine-Induced Cell Death

**DOI:** 10.3390/ijms26167768

**Published:** 2025-08-12

**Authors:** Esra Münire Cüce-Aydoğmuş, Pınar İyiol, Günseli Ayşe İnhan-Garip

**Affiliations:** 1 Biophysics Department, School of Medicine, T.C. Maltepe University, Istanbul 34857, Turkey; 2 Biophysics Department, School of Medicine, T.C. Marmara University, Istanbul 34854, Turkey; pinariyiol28@gmail.com (P.İ.); aysein9@yahoo.com (G.A.İ.-G.)

**Keywords:** voltage-gated K channels, membrane potential, intracellular Ca concentration, ion channel blockers, breast cancer cell line

## Abstract

Cancer has recently been proposed as a type of channelopathy due to the aberrant expression of various ion channels. Voltage-gated potassium (K^+^) channels (VGKCs) are notably upregulated during tumor proliferation, while voltage-gated sodium (Na^+^) channels are predominantly associated with the invasive stage of cancer progression. Among these, the Kv10.1 channel has been found to be overexpressed in breast cancer, making it a promising therapeutic target. 4-Aminopyridine (4-AP), a non-selective voltage-gated potassium channel blocker, has emerged as a potential novel agent for breast cancer treatment. In this study, we aimed to elucidate the mechanism of action of 4-aminopyridine in breast cancer cells. To investigate the involvement of various cell death pathways, cycloheximide (CHX) (a paraptosis inhibitor), Z-VAD-FMK (a pan-caspase inhibitor), and 2-Aminoethoxydiphenyl borate (2-APB) (a phosphoinositide 3-kinase [PI3K] inhibitor) were employed. Experiments were conducted using the MCF-7 human breast cancer cell line and the L929 mouse fibroblast cell line as a healthy control. Assessments included cell viability assays, intracellular calcium (Ca^2+^) and K^+^ concentration measurements, and plasma membrane potential analysis. Our findings aim to contribute to the understanding of the therapeutic potential and cellular effects of VGKC blockers, particularly 4-aminopyridine, in breast cancer treatment strategies.

## 1. Introduction

Ion channels play crucial roles in tumor pathophysiology, contributing to the regulation of membrane potential, cell cycle progression, cellular osmolarity, motility, invasion, migration, and proliferation [1,2]. Among these, potassium (K^+^) channels—encoded by approximately 77 genes—represent the most diverse and abundant group in excitable cells. These channels are essential for maintaining ionic homeostasis, selectively permitting potassium ion flux across membranes in response to various environmental stimuli while restricting the movement of other ions.

Based on their activation mechanisms, ion channels are generally classified into voltage-gated, ligand-gated, and mechanically gated types. The term “ion channel” often refers specifically to voltage-gated channels, whose activity is predominantly regulated by membrane potential. These channels are named according to the ions that most readily permeate them.

Recently, cancer has increasingly been considered a channelopathy, and there is growing interest in targeting ion channel modulation as a novel therapeutic approach, particularly in breast cancer [1].

Potassium channel proteins are involved in a wide range of physiological and pathological processes [2]. Notably, dysregulation and abnormal expression of potassium channels have been observed in several types of cancer, including breast [3], colorectal [4], prostate [5], lung [6], liver [7], and glioma [8]. Several subtypes, such as *KCNN4* [9], *KCNA1* [10], Kv11.1 [11], *KCNK9* [11,12], *KCNE1* [13], and *GIRK1* [14], have been implicated in the malignant transformation and progression of breast cancer. Additionally, KCNK6 has been reported to be overexpressed in both breast cancer [15] and thyroid carcinoma [16].

Hou et al. demonstrated that *KCNK6* expression is elevated in breast cancer cells, leading to weakened cell adhesion [17]. Pharmacological inhibition of this channel enhanced cellular adhesion and biophysical function, suggesting that upregulation of *KCNK6* may facilitate cancer cell proliferation, migration, and invasion. Similarly, Sun et al. reported that *KCNK1* is overexpressed in breast cancer and associated with poor prognosis [18]. Silencing of *KCNK1* reduced cell proliferation and migration while enhancing sensitivity to paclitaxel chemotherapy.

In MCF-7 breast cancer cells, the “membrane potential” model proposed by Wonderlin et al. suggests that both proliferation and cell cycle progression are tightly linked to K^+^ channel activity [19]. Inhibition of these channels causes membrane depolarization, which in turn suppresses cell proliferation. Electrophysiological studies have revealed that at least six distinct K^+^ currents—differing in their dependence on voltage, intracellular Ca^2+^, and ATP—are present in MCF-7 cells [20,21,22,23].

However, the precise mechanisms by which non-selective K^+^ channel blockers such as 4-aminopyridine (4-AP) induce cell death in breast cancer cells remain unclear. 4-AP blocks potassium channels, leading to cell depolarization and calcium influx. Due to this property, it enhances neurotransmitter release by increasing Ca^2+^ influx in the presynaptic region and accelerates axonal conduction, making it useful in the treatment of central and peripheral nervous system disorders such as Multiple Sclerosis (MS), spinal cord injury (SCI), botulism, and Lambert–Eaton syndrome [24].

Given 4-AP’s action as a potassium channel blocker, the altered expression of potassium channels in cancer cells, and the effects of channel blockade on cell proliferation [13,25,26], studies have shown that 4-AP inhibits cancer cell proliferation and induces apoptosis [27]. Since 4-AP is known to increase intracellular calcium levels, the vacuolization observed in our previous experimental observations (unpublished microscopy data) prompted us to further investigate whether 4-AP induces paraptosis-like cell death.

Paraptosis is distinguished from apoptosis by the absence of nuclear fragmentation, apoptotic body formation, chromatin condensation, and caspase-dependent cell death. Instead, paraptotic cells exhibit extensive cytoplasmic vacuolation, often associated with the dilation of the endoplasmic reticulum (ER) and mitochondria. Treatment with cycloheximide (CHX) has been shown to suppress vacuolation-mediated cell death, suggesting that active protein synthesis is required for the execution of paraptosis. Various mechanisms have been implicated in the induction of paraptosis, including overexpression of insulin-like growth factor 1 receptor (IGF1R), proteasome inhibition, ER stress, reactive oxygen species (ROS) generation, mitochondrial Ca^2+^ influx, and ion channel activation [28].

In this study, we hypothesized that blocking voltage- gated K^+^ channels (VGKCs) with 4-AP alters membrane potential and calcium signaling, leading to regulated cell death. We employed pharmacological inhibitors—including CHX, Z-VAD-FMK, and 2-Aminoethoxydiphenyl borate (2-APB) —to dissect the involvement of apoptotic and calcium-dependent cell death pathways. The effects of 4-AP on cell viability, membrane potential, and intracellular calcium concentration were systematically evaluated.

## 2. Results

### 2.1. Determination of IC_50_ Values and the Investigation of Cell Death Mechanisms Induced by 4-AP in L929 and MCF-7 Cell Lines

**Figure 1:**
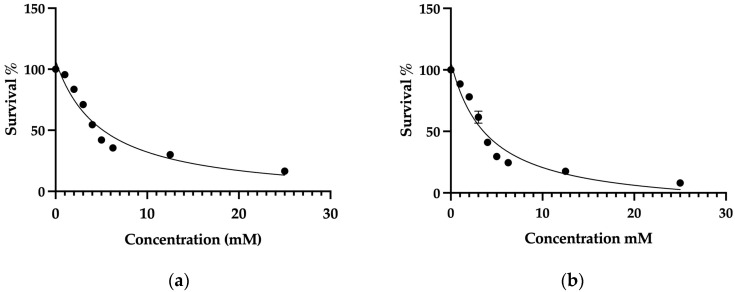
Determination of the IC_50_ values of 4-AP in L929 and MCF-7 cell lines. Concentrations of 25 mM, 12.5 mM, and 6.25 mM were used as the highest doses. Since the expected IC_50_ range was between 1 and 5 mM, finer dose increments of 1 mM were applied within this range. Cell viability was assessed using the trypan blue exclusion method with a hemocytometer. Following viability measurements, inhibitor vs. response curves were plotted to calculate IC_50_ values. The IC_50_ value of 4-AP was determined to be 5 mM in L929 cells (**a**) and 4 mM in MCF-7 cells (**b**). Data represent mean ± SEM from more than three independent experiments (*n* ≥ 3).

The IC_50_ values of 4-AP for L929 (healthy fibroblast) and MCF-7 (breast cancer) cell lines were determined using the trypan blue exclusion method with a hemocytometer. A concentration range of 1–25 mM was used to determine the IC_50_ values. The IC_50_ value of 4-AP was found to be 4 mM for MCF-7 cells and 5 mM for L929 cells (Figure 1a,b). The higher IC_50_ observed in L929 cells indicates lower sensitivity to 4-AP compared to MCF-7 cells. L929 cells, which do not exhibit overexpression of voltage-gated potassium channels, were selected as the healthy control line. The considerable difference in IC_50_ values between the two cell lines suggests that 4-AP exhibits selective cytotoxicity, potentially related to differential ion channel expression.

In subsequent experiments, the effects of 4-AP on cell death mechanisms were investigated using CHX, z-VAD-FMK, and 2-APB based on concentrations previously reported in the literature.

**Figure 2:**
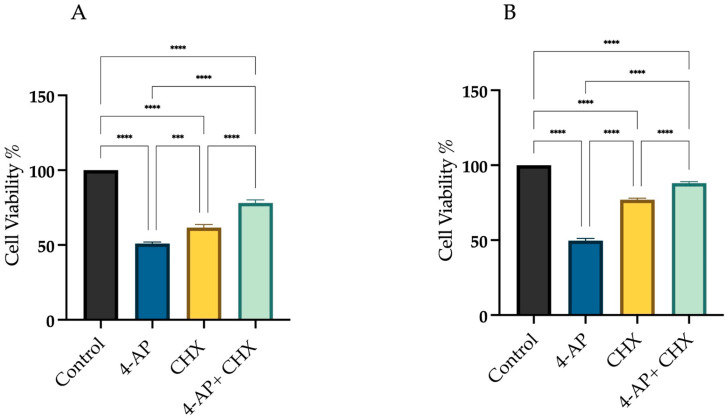
Assessment of cell viability following CHX and 4-AP treatments. Cells were treated with the IC_50_ concentration of 4-AP, and cell viability was assessed using the trypan blue exclusion method with a hemocytometer. For CHX treatment, a 20 µM dose was selected. Cells were pre-incubated with CHX for 1 h prior to 4-AP exposure. CHX pre-treatment resulted in increased cell viability in both the L929 (**A**) and MCF-7 (**B**) cell lines. *** *p* < 0.001, and **** *p* < 0.0001, *n* ≥ 3.

Figure 2 presents the following experimental conditions: (i) the change in cell viability following 24 h exposure to 4-AP at its IC_50_, (ii) the viability reduction observed after 24 h treatment with 20 µM CHX alone, and (iii) the effects of a 1 h CHX pre-treatment followed by a 24 h 4-AP exposure. At this dose, cell viability was 61.6% ± 2.4 for L929 and 77% ± 1.5 for MCF-7. Cells were pre-treated with CHX for 1 h and then treated with 4-AP for 24 h. After the combined treatment, cell viability increased to 78% ± 2.2 in L929 and 88% ± 1.2 in MCF-7 cells (Figure 2A,B). Notably, a 1 h CHX pre-treatment would not induce cytotoxicity, unlike extended CHX treatments (>6–7 h), which are known to significantly reduce viability [29,30]. Therefore, we conclude that the 1 h CHX regimen is non-cytotoxic and specifically serves to block protein synthesis-dependent paraptotic pathways, thereby protecting cells from anticipated 4-AP-induced paraptotic death. If 4-AP-induced cytotoxicity were solely due to paraptosis, then these viability levels would be expected to be even higher, suggesting the involvement of other cell death mechanisms.

**Figure 3:**
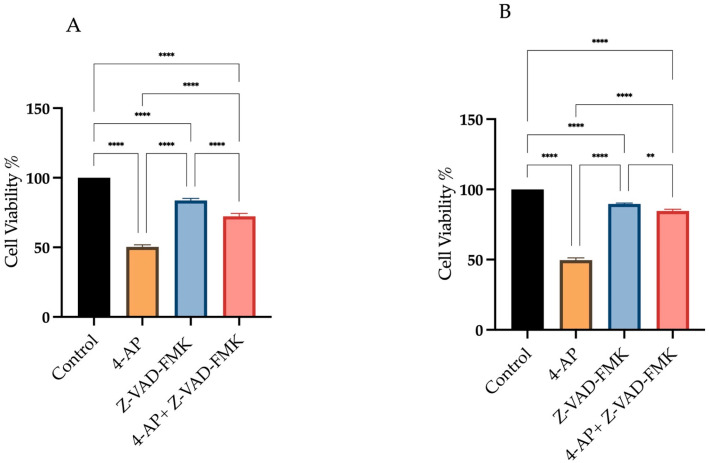
Assessment of cell viability following Z-VAD-FMK and 4-AP treatments. Cells were treated with 50 µM Z-VAD-FMK, either alone or in combination with 4-AP. Cell viability was assessed using the trypan blue exclusion method with a hemocytometer. Treatment with Z-VAD-FMK alone resulted in a slight decrease in cell viability compared to the control group. However, pre-treatment with Z-VAD-FMK for 1 h prior to 4-AP exposure significantly reduced cell viability in both L929 (**A**) and MCF-7 (**B**) cell lines. ** *p* < 0.01, **** *p* < 0.0001, *n* ≥ 3.

To determine the contribution of apoptosis to 4-AP-induced cell death, we used Z-VAD-FMK, a broad-spectrum pan-caspase inhibitor. As shown in Figure 3, we assessed cell viability under three conditions: (i) after 24 h treatment with 4-AP at its IC_50_ concentration, (ii) following a 24 h exposure to 50 µM Z-VAD-FMK alone, and (iii) after a 1 h pre-treatment with Z-VAD-FMK, followed by 24 h incubation with 4-AP. Treatment with Z-VAD-FMK alone resulted in cell viabilities of 83.6% ± 1.9 for L929 and 91% ± 0.9 for MCF-7. Following 1 h pre-treatment with Z-VAD-FMK, cells were incubated with 4-AP for 24 h. Under these conditions, cell viability decreased to 72.3% ± 2.4 in L929 and 85% ± 1.2 in MCF-7 (Figure 3A,B). Despite effective caspase inhibition, a significant reduction in cell viability was still observed in the combination group. This suggests that 4-AP–induced cytotoxicity is not solely mediated by caspase-dependent apoptosis but rather involves non-apoptotic, caspase-independent pathways, such as paraptosis or necrosis.

These findings align with previous reports demonstrating that Z-VAD-FMK is insufficient to fully prevent cell death in certain contexts, where cells may switch to alternative death mechanisms in the absence of caspase activity [31,32,33].

**Figure 4:**
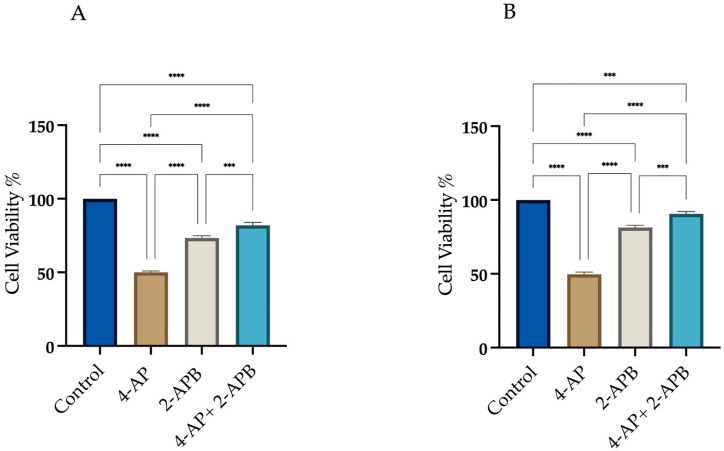
Assessment of cell viability following 2-APB and 4-AP treatments. Cells were treated with 10 µM 2-APB, either alone or in combination with 4-AP. Cell viability was assessed using the trypan blue exclusion method with a hemocytometer. Pre-treatment with 2-APB for 1 h increased cell viability in both L929 (**A**) and MCF-7 (**B**) cell lines. *** *p* < 0.001, and **** *p* < 0.0001, *n* ≥ 3.

To assess the role of calcium signaling in 4-AP-induced cell death, 2-APB, an IP3 receptor inhibitor, was employed at a concentration of 10 µM. As shown in Figure 4, we assessed cell viability under three conditions: (i) after 24 h treatment with 4-AP at its IC_50_ concentration, (ii) following a 24 h exposure to 10 µM 2-APB alone, and (iii) after a 1 h pre-treatment with 2-APB, followed by 24 h incubation with 4-AP.

Treatment with 2-APB alone resulted in cell viabilities of 73.3% ± 1.9 for L929 and 73% ± 2.1 for MCF-7 with respect to the control. Following 1 h pre-treatment with 2-APB and subsequent incubation with 4-AP for 24 h, cell viability increased to 82% ± 2.5 for L929 and 92% ± 2.0 for MCF-7 (Figure 4A,B). These findings suggest that IP3-mediated calcium signaling plays a significant role in 4-AP-induced cytotoxicity. The high cell viability observed after combined 2-APB and 4-AP treatment implies that inhibition of calcium signaling may prevent both apoptosis and paraptosis. Yoon et al. employed the IP_3_ receptor antagonist 2-APB to evaluate the role of calcium release from the endoplasmic reticulum in paraptotic cell death and reported a reduction in cell death [34]. Following a similar rationale, we utilized 2-APB in our experiments. Pre-treatment with 2-APB resulted in an increase in cell viability.

### 2.2. Assessment of Intracellular Calcium Changes Following Drug Treatments

**Figure 5:**
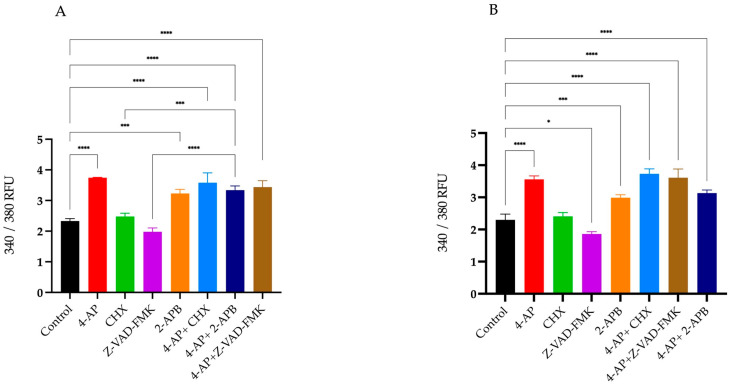
Percentage changes in intracellular Ca^2+^ levels following treatments. Intracellular calcium concentrations were measured using the Fura-2 fluorescent dye. Percentage changes in Ca^2+^ levels were calculated relative to the untreated control group. Treatment with 4-AP and its combinations increased intracellular Ca^2+^ levels. CHX and Z-VAD-FMK alone did not induce significant increases, whereas 2-APB alone elevated intracellular Ca^2+^ concentrations in both L929 (**A**) and MCF-7 (**B**) cell lines. * *p* < 0.05, *** *p* < 0.001, and **** *p* < 0.0001, *n* ≥ 3. Non-significant results are not indicated in the figure.

After drug treatments, changes in intracellular calcium levels were measured using the Screen Quest Fura-2 AM no-wash calcium assay kit. Fura-2 crosses the cell membrane and enters the cytoplasm. When Fura-2 binds to free calcium ions, its fluorescence emission wavelength shifts towards the blue region. When agonists or other signals stimulate the cell, receptor activation triggers the release of calcium, leading to an increase in the fluorescent intensity of Fura-2. For analysis of intracellular Ca^2+^ changes, Fura-2 measurements were taken immediately after drug addition for 20 h. The results at 20th hour are presented as a histogram (Figure 5A,B).

The percentages of changes in intracellular Ca^2+^ concentration and cell viabilities after treatments are presented in the table relative to the control group (Table 1). Briefly, 4-AP and its combinations caused an increase in intracellular Ca^2+^ concentration. CHX induced a slight increase, whereas Z-VAD-FMK led to a decrease in intracellular Ca^2+^ levels. Although 2-APB is an IP_3_ receptor blocker, its treatment resulted in an unexpected increase in intracellular Ca^2+^ concentration. 2-APB both alone and in combination with 4-AP increased intracellular Ca^2+^ levels. The combination effect of 4-AP and 2-APB can be explained by the blockade of VGKCs by 4-AP, which causes membrane depolarization. This depolarization likely opens voltage-gated calcium channels (VGCCs), leading to an increase in intracellular Ca^2+^ concentration.

### 2.3. Membrane Potential Measurements: Effect of Drug Treatments on Membrane Polarization

Changes in membrane potential induced by drug treatments were measured using the DiBAC_4_(3) fluorescent dye. DiBAC_4_(3) is a hydrophobic dye that easily passes through the cell membrane, enabling detection of membrane potential alterations. In the absence of stimulation, the negative charge on the cytoplasmic side of the membrane prevents the ionized dye from entering the cell. Upon membrane depolarization triggered by stimulation, the dye binds to intracellular proteins and lipids, leading to an increase in fluorescence intensity. Conversely, during hyperpolarization, the fluorescence intensity of the dye decreases.

To determine changes in membrane potential following the inhibition of 4-AP-induced cell death, drugs and their combinations with 4-AP were administered, and DiBAC_4_(3) fluorescence measurements were performed using a microplate reader 15 min after treatments for 24 h.

**Figure 6:**
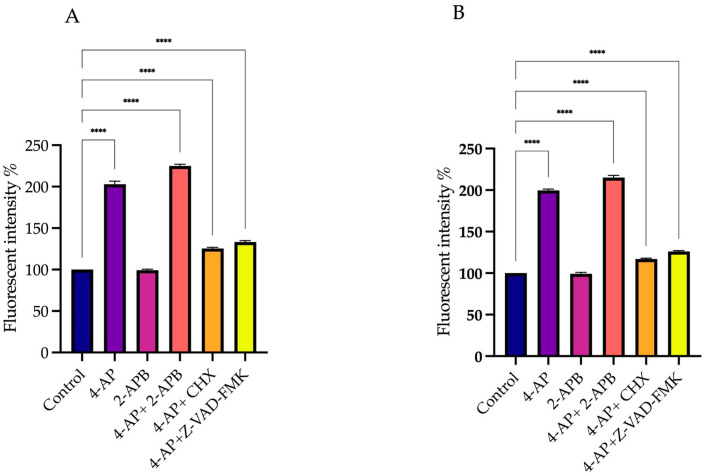
Effect of drug treatments on membrane polarization. To assess the effects of elevated intracellular Ca^2+^ levels on membrane potential, DiBAC_4_(3) fluorescent dye was used. Since CHX and Z-VAD-FMK did not induce significant increases in intracellular Ca^2+^ levels, they were excluded from DiBAC_4_(3) measurements. Treatment with 4-AP and its combinations increased membrane depolarization, with the most pronounced effect observed following co-treatment with 2-APB. In contrast, 2-APB alone did not cause substantial depolarization in either L929 (**A**) or MCF-7 (**B**) cell lines. **** *p* < 0.0001, *n* ≥ 3. Non-significant results are not indicated in the figure.

Measurements were taken in a time-dependent manner. In the L929 cell line, 4-AP treatment induced membrane depolarization. Since 4-AP blocks VGKCs, this blockade results in membrane depolarization. The combination of 4-AP and 2-APB also led to depolarization (Figure 6A). The depolarization caused by 4-AP likely activated VGCCs, enhancing calcium influx and resulting in a greater degree of depolarization than 4-AP treatment alone. Combinations of 4-AP with CHX or Z-VAD-FMK also induced membrane depolarization; however, the depolarization levels were lower compared to 4-AP alone or the 4-AP + 2-APB combination. This difference may be attributed to intracellular accumulation of potassium ions without additional VGCC activation. Treatment with 2-APB alone did not induce membrane depolarization.

In the MCF-7 cell line, a similar pattern was observed. 4-AP treatment alone induced membrane depolarization, and its combination with 2-APB enhanced this effect. However, 4-AP combined with CHX or Z-VAD-FMK caused only a slight depolarization of the membrane (Figure 6B).

## 3. Discussion

In this study, we demonstrated that 4-aminopyridine, a known potassium channel blocker used in neurological disorders, induces cell death in MCF-7 breast cancer cells through both apoptotic and paraptotic pathways. Despite the development of chemotherapeutic agents, resistance to apoptosis remains a major limitation in effective cancer treatment [35]. Therefore, triggering alternative cell death mechanisms, such as paraptosis, has become increasingly relevant in overcoming therapeutic resistance.

Our results showed that treatment with 4-AP induced vacuolization and cell swelling in MCF-7 cells, phenomena commonly associated with paraptotic or paraptosis-like cell death. This observation aligns with previous reports indicating that paraptosis is characterized by cytoplasmic vacuole formation, swelling of the endoplasmic reticulum (ER) and mitochondria, and reliance on protein synthesis [36,37]. The increased cell viability upon treatment with the protein synthesis inhibitor CHX, which raised MCF-7 survival rates from 53% to 88%, further suggests involvement of a paraptotic mechanism [38]. Nonetheless, we acknowledge that our study does not provide direct morphological or biochemical evidence to definitively confirm paraptosis. Therefore, additional targeted investigations are warranted to clarify the precise mode of cell death induced by 4-AP in MCF-7 cells.

Furthermore, we assessed whether apoptotic pathways were simultaneously involved in the 4-AP-induced cell death process. The application of the pan-caspase inhibitor z-vad-fmk significantly increased survival rates in both MCF-7 and L929 cells, suggesting that apoptosis also contributes to the observed cytotoxicity. These findings align with other studies reporting simultaneous activation of multiple cell death pathways in cancer cells exposed to various compounds, including honokiol, celastrol, and iturin A [39,40,41].

Interestingly, although caspase-9 activity is reported to be required for paraptosis [42], Sperandio et al. demonstrated that paraptosis is not inhibited by z-vad-fmk, indicating mechanistic complexity [38]. Our findings that z-vad-fmk increases survival suggest partial overlap or co-activation of apoptotic and paraptotic pathways. To delineate the sequence and dominance of these pathways, future experiments using selective caspase-9 inhibitors and paraptosis-specific blockers such as AIP1/Alix are needed.

Another key observation in our study was the intracellular calcium (Ca^2+^) influx following 4-AP treatment. Using Fura-2-based analyses, we confirmed that 4-AP induced significant increases in intracellular Ca^2+^ levels. Pre-incubation with the IP3 receptor antagonist 2-APB improved survival rates (92% in MCF-7 cells), indicating that ER-derived Ca^2+^ release contributes to 4-AP-induced cell death. These findings are consistent with the notion that disruption of Ca^2+^ homeostasis is central to both apoptosis and paraptosis [43].

However, we also observed that 2-APB alone induced Ca^2+^ elevation greater than 4-AP. This may be explained by its known activation of Orai3 channels in estrogen receptor-positive breast cancer cells, as previously reported [44,45]. Therefore, the context-dependent action of 2-APB and its concentration-specific effects on calcium channels must be carefully considered in interpreting these data [46].

**Figure 7:**
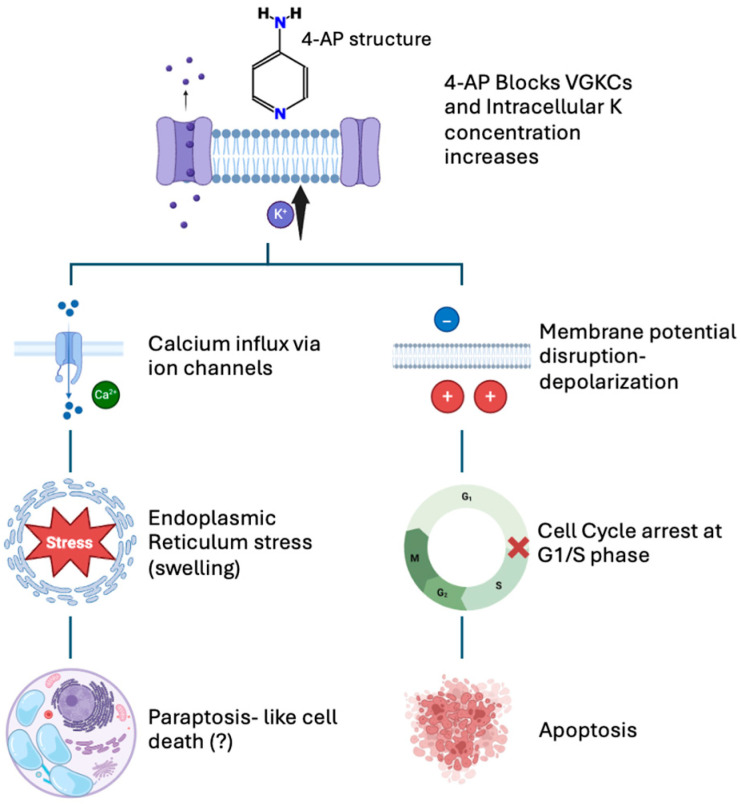
Proposed mechanism of 4-AP-induced cell death in MCF-7 breast cancer cells. 4-AP blocks voltage-gated potassium (K^+^) channels, causing intracellular accumulation of K^+^ ions and membrane depolarization (top panel). Both the increased intracellular K^+^ concentration and membrane depolarization contribute to enhanced calcium (Ca^2+^) influx into the cell. Elevated Ca^2+^ levels trigger endoplasmic reticulum (ER) stress, suggesting paraptosis-like cell death characterized by cytoplasmic vacuolization and organelle swelling (left pathway). Additionally, membrane depolarization causes cell cycle arrest at the G1/S phase, inducing apoptosis (right pathway). This schematic summarizes the dual pathways underlying 4-AP-mediated cytotoxic effects.

The observed ER and mitochondrial changes, combined with altered calcium signaling, support the idea that 4-AP simultaneously triggers both mitochondrial-mediated apoptosis and ER-related paraptosis. Nevertheless, temporal dynamics remain unclear. Confocal microscopy-based kinetic analyses of intracellular Ca^2+^ distribution, in combination with selective pathway inhibitors, will be crucial to clarify the sequence of events. Based on the collective findings presented above, a schematic model is proposed to illustrate the dual mechanism of 4-AP-induced cytotoxicity in MCF-7 cells (Figure 7).

In conclusion, our findings suggest that 4-AP induces cancer cell death via dual activation of paraptotic and apoptotic pathways. This dual action may overcome apoptosis resistance in certain cancer types. Although 4-AP is unlikely to serve as a standalone anticancer agent, its ability to modulate ion channel activity and disrupt Ca^2+^ homeostasis makes it a promising candidate for combination therapy with conventional chemotherapeutics, as demonstrated by our previous findings that 4-AP in combination with paclitaxel increased cell death in both MCF-7 and MDA-MB-231 breast cancer cell lines [47].

## 4. Materials and Methods

### 4.1. Cell Lines and the Growth Condition

The breast cancer cell line MCF-7 (ATCC HTB 22) and healthy mouse fibroblast cell line L929 (ATCC CCL-1) were used in this study. Cells were grown in DMEM medium containing L-glutamine (Capricorn Scientific GmbH, Ebsdorfergrund, Germany) supplemented with 10% Fetal Bovine Serum (FBS; Capricorn Scientific GmbH, Ebsdorfergrund, Germany) and 1% penicillin/streptomycin (Capricorn Scientific GmbH, Ebsdorfergrund, Germany) in a humidified atmosphere containing 5% CO_2_ at 37 °C.

### 4.2. Cell Viability and Cytotoxicity of 4-AP, Z-VAD-FMK, CHX, 2-APB: Determination of IC50 Values

4-AP, Z-VAD-FMK, CHX, and 2-APB (Sigma Aldrich, St. Louis, MO, USA) stock solutions were prepared by dissolving in DMSO (Sigma Aldrich, St. Louis, MO, USA) and stored at −20 °C. Growth medium was used for preparing the desired concentration from the stock solution. The final concentration of DMSO was 0.01%, and the control groups were with and without DMSO. From a cell survival point of view, no difference was observed between the control without DMSO and the control with DMSO.

Determinations of viability and IC50 values were performed by the trypan blue exclusion method using a hemocytometer. MCF-7 and L929 cells were seeded on 6-well plates, with cell numbers of 1.8 × 10^5^ cells/well in 3 mL growth medium, and incubated overnight in a humidified atmosphere containing 5% CO_2_ at 37 °C. After incubation, cells were treated with different concentrations of 4-AP, Z-VAD-FMK, CHX, and 2-APB and incubated for 24 h and then counted with a hemocytometer. Results were compared with control groups.

### 4.3. Determination of Intracellular Ca^2+^ Concentration

Activation of calcium channels or G protein-coupled receptors leads to changes in intracellular calcium concentrations, which can be homogeneously detected using the Fura-2 calcium indicator. Once inside the cell, esterases cleave the lipophilic and blocking groups of Fura-2, trapping the negatively charged fluorescent dye within the cell.

Changes in intracellular Ca^2+^ concentration were measured using the Screen Quest™ Fura-2 No-Wash Calcium Assay Kit (AAT Bioquest Inc., Pleasanton, CA, USA) with a microplate reader (Synergy H1, BioTek Instruments, Winooski, VT, USA). Fura-2 AM was dissolved in DMSO and used according to the manufacturer’s protocol.

Cells were seeded in black 96-well plates at a density of 1 × 10^4^ cells per well in 100 µL of growth medium and incubated overnight. Intracellular calcium levels were detected by measuring fluorescence at dual excitation wavelengths of 340 nm and 380 nm and an emission wavelength of 510 nm.

Fluorescence measurements were performed for 90 min immediately following drug addition to monitor dynamic calcium responses.

### 4.4. Measurement of Transmembrane Potential Using DiBAC_4_(3)

The transmembrane potential was measured using the fluorescent dye Bis-(1,3-dibutyl barbituric acid) trimethine oxonol (DiBAC_4_(3); AAT Bioquest Inc., Pleasanton, CA, USA) with a microplate reader (Synergy H1, BioTek Instruments, Winooski, VT, USA). DiBAC_4_(3) is a hydrophobic, slow-response dye that passively diffuses across the cell membrane. Upon incubation, the dye migrates from the extracellular aqueous medium into the lipid bilayer due to its hydrophobic nature. Unlike cationic carbocyanine dyes, DiBAC_4_(3) is not absorbed by mitochondria and is primarily selective for changes in membrane potential.

The dye was prepared at a stock concentration of 20 mM in spectroscopic-grade DMSO. Cells were seeded in black 96-well plates at a density of 5 × 10^4^ cells per well in 100 µL of growth medium and incubated overnight. After incubation, the medium was removed and replaced with 100 µL of HBSS. Drug treatments were applied, and cells were incubated for 2 h. Following drug treatment, DiBAC_4_(3) was added to each well to reach a final concentration of 2 µM in 100 µL HBSS, and the cells were incubated for 30 min at room temperature.

To remove excess dye, cells were washed with HBSS, and drug treatments were repeated. Fluorescence measurements were performed in HBSS medium with an excitation wavelength of 540 nm and an emission wavelength of 590 nm. Fluorescence intensity was recorded every 30 min over a 24 h period to monitor dynamic changes in membrane potential.

### 4.5. Statistical Analyzes

Data in this study were obtained from at least three independent experiments. Results are presented as mean ± standard deviation (SD) in the figures. Statistical analyses were conducted using a two-tailed unpaired Student’s *t*-test for comparisons between two groups and one-way analysis of variance (ANOVA) for comparisons among multiple groups; post hoc analysis was performed using Tukey’s test. For all analyses, *n* ≥ 3. Differences were considered statistically significant at *p* < 0.05 and are indicated as follows: * *p* < 0.05, ** *p* < 0.01, *** *p* < 0.001, and **** *p* < 0.0001.

## Figures and Tables

**Table 1:** The percentages of changes in intracellular Ca^2+^ concentration and cell viabilities.

Treatments	L929		MCF-7	
	Cell Viability	The Changes in Intracellular Ca^2+^	Cell Viability	The Changes in Intracellular Ca^2+^
4-AP	50.3% ± 2.3	60% ± 0.19	49.6% ± 2.9	55% ± 0.13
CHX	61.6% ± 2.4	6% ± 0.20	77% ± 1.5	4% ± 0.11
Z-VAD-FMK	83.6% ± 1.9	−16% ± 0.30	91% ± 0.9	−19% ± 0.10
2-APB	73.3% ± 1.9	38% ± 0.10	73% ± 2.1	30% ± 0.11
4-AP+CHX	78% ± 2.2	53% ± 0.37	88% ± 1.2	62% ± 0.17
4-AP+Z-VAD-FMK	72.3% ± 2.4	47% ± 0.10	85% ± 1.2	57% ± 0.26
4-AP+2-APB	82% ± 2.5	42% ± 0.10	92% ± 2.0	36% ± 0.12

## Data Availability

The data that support the findings of this study are available from the corresponding author upon reasonable request.

## Abbreviations

The following abbreviations are used in this manuscript:

4-AP	4-Aminopyridine
2-APB	2-Aminoethoxydiphenyl borate
CHX	Cycloheximide
DiBAC_4_(3)	Bis-(1,3-dibutylbarbituric acid) trimethine oxonol
ER	Endoplasmic reticulum
VGKC	Voltage-gated potassium channel
VGCC	Voltage-gated calcium channel
IP3	Inositol 1,4,5-trisphosphate
MS	Multiple Sclerosis
SCI	spinal cord injury
DMEM	Dulbecco’s Modified Eagle’s Medium
FBS	Fetal bovine serum
DMSO	Dimethyl sulfoxide
Orai3	Orai Calcium Release-Activated Calcium Modulator 3

## References

[1] Pardo L.A., Stühmer W. (2014). The Roles of K^+^ Channels in Cancer. Nat. Rev. Cancer.

[2] Schwab A., Fabian A., Hanley P.J., Stock C. (2012). Role of Ion Channels and Transporters in Cell Migration. Physiol. Rev..

[3] Ko J.-H., Ko E.A., Gu W., Lim I., Bang H., Zhou T. (2013). Expression Profiling of Ion Channel Genes Predicts Clinical Outcome in Breast Cancer. Mol. Cancer.

[4] Ishaque N., Abba M.L., Hauser C., Patil N., Paramasivam N., Huebschmann D., Leupold J.H., Balasubramanian G.P., Kleinheinz K., Toprak U.H. (2018). Whole Genome Sequencing Puts Forward Hypotheses on Metastasis Evolution and Therapy in Colorectal Cancer. Nat. Commun..

[5] Rose A.M., Krishan A., Chakarova C.F., Moya L., Chambers S.K., Hollands M., Illingworth J.C., Williams S.M.G., McCabe H.E., Shah A.Z. (2018). MSR1 Repeats Modulate Gene Expression and Affect Risk of Breast and Prostate Cancer. Ann. Oncol..

[6] Zhang X., Zhang L., Lin B., Chai X., Li R., Liao Y., Deng X., Liu Q., Yang W., Cai Y. (2017). Phospholipid Phosphatase 4 Promotes Proliferation and Tumorigenesis, and Activates Ca^2+^-Permeable Cationic Channel in Lung Carcinoma Cells. Mol. Cancer.

[7] Wang H., Zou L., Ma K., Yu J., Wu H., Wei M., Xiao Q. (2017). Cell-Specific Mechanisms of TMEM16A Ca^2+^-Activated Chloride Channel in Cancer. Mol. Cancer.

[8] Huang X., He Y., Dubuc A.M., Hashizume R., Zhang W., Reimand J., Yang H., Wang T.A., Stehbens S.J., Younger S. (2015). EAG2 Potassium Channel with Evolutionarily Conserved Function as a Brain Tumor Target. Nat. Neurosci..

[9] Steudel F.A., Mohr C.J., Stegen B., Nguyen H.Y., Barnert A., Steinle M., Beer-Hammer S., Koch P., Lo W., Schroth W. (2017). SK4 Channels Modulate Ca^2+^ Signalling and Cell Cycle Progression in Murine Breast Cancer. Mol. Oncol..

[10] Lallet-Daher H., Wiel C., Gitenay D., Navaratnam N., Augert A., Le Calvé B., Verbeke S., Carling D., Aubert S., Vindrieux D. (2013). Potassium Channel KCNA1 Modulates Oncogene-Induced Senescence and Transformation. Cancer Res..

[11] Breuer E.-K., Fukushiro-Lopes D., Dalheim A., Burnette M., Zartman J., Kaja S., Wells C., Campo L., Curtis K.J., Romero-Moreno R. (2019). Potassium Channel Activity Controls Breast Cancer Metastasis by Affecting β-Catenin Signaling. Cell Death Dis..

[12] Sun H., Luo L., Lal B., Ma X., Chen L., Hann C.L., Fulton A.M., Leahy D.J., Laterra J., Li M. (2016). A Monoclonal Antibody against KCNK9 K^+^ Channel Extracellular Domain Inhibits Tumour Growth and Metastasis. Nat. Commun..

[13] Becchetti A. (2011). Ion Channels and Transporters in Cancer. 1. Ion Channels and Cell Proliferation in Cancer. Am. J. Physiol.-Cell Physiol..

[14] Stringer B.K., Cooper A.G., Shepard S.B. (2001). Overexpression of the G-Protein Inwardly Rectifying Potassium Channel 1 (GIRK1) in Primary Breast Carcinomas Correlates with Axillary Lymph Node Metastasis. Cancer Res..

[15] Williams S., Bateman A., O’Kelly I. (2013). Altered Expression of Two-Pore Domain Potassium (K2P) Channels in Cancer. PLoS ONE.

[16] Lin X., Wu J.-F., Wang D.-M., Zhang J., Zhang W.-J., Xue G. (2020). The Correlation and Role Analysis of KCNK2/4/5/15 in Human Papillary Thyroid Carcinoma Microenvironment. J. Cancer.

[17] Hou X., Tang L., Li X., Xiong F., Mo Y., Jiang X., Deng X., Peng M., Wu P., Zhao M. (2021). Potassium Channel Protein KCNK6 Promotes Breast Cancer Cell Proliferation, Invasion, and Migration. Front. Cell Dev. Biol..

[18] Sun X., Li Y., Lan H., Jiang T., Wan X., Cheng Y. (2023). Identification of KCNK1 as a Potential Prognostic Biomarker and Therapeutic Target of Breast Cancer. Pathol. Res. Pract..

[19] Wonderlin W.F., Woodfork K.A., Strobl J.S. (1995). Changes in membrane potential during the progression of MCF-7 human mammary tumor cells through the cell cycle. J. Cell Physiol..

[20] Wegman E.A., Young J.A., Cook D.I. (1991). A 23-pS Ca^2+^-Activated K^+^ Channel in MCF-7 Human Breast Carcinoma Cells: An Apparent Correlation of Channel Incidence with the Rate of Cell Proliferation. Pflügers Arch..

[21] Klimatcheva E., Wonderlin W.F. (1999). An ATP-Sensitive K^+^ Current That Regulates Progression through Early G1 Phase of the Cell Cycle in MCF-7 Human Breast Cancer Cells. J. Membr. Biol..

[22] Ouadid-Ahidouch H., Ahidouch A. (2008). K^+^ Channel Expression in Human Breast Cancer Cells: Involvement in Cell Cycle Regulation and Carcinogenesis. J. Membr. Biol..

[23] Ouadid-Ahidouch H., Roudbaraki M., Delcourt P., Ahidouch A., Joury N., Prevarskaya N. (2004). Functional and Molecular Identification of Intermediate-Conductance Ca^2+^-Activated K^+^ Channels in Breast Cancer Cells: Association with Cell Cycle Progression. Am. J. Physiol.-Cell Physiol..

[24] van der Bruggen M.A., Huisman H.B., Beckerman H., Bertelsmann F.W., Polman C.H., Lankhorst G.J. (2001). Randomized Trial of 4-Aminopyridine in Patients with Chronic Incomplete Spinal Cord Injury. J. Neurol..

[25] Lastraioli E. (2018). Potassium channels in breast cancer. Ann. Breast Cancer.

[26] Villalonga N., Ferreres J.C., Argilés J.M., Condom E., Felipe A. (2007). Potassium Channels Are a New Target Field in Anticancer Drug Design. Recent Pat. Anticancer Drug Discov..

[27] Kim J.A., Kang Y.S., Jung M.W., Kang G.H., Lee S.H., Lee Y.S. (2000). Ca^2+^ Influx Mediates Apoptosis Induced by 4-Aminopyridine, a K^+^ Channel Blocker, in HepG2 Human Hepatoblastoma Cells. Pharmacology.

[28] Hanson S., Dharan A., V. J.P., Pal S., Nair B.G., Kar R., Mishra N. (2023). Paraptosis: A unique cell death mode for targeting cancer. Front. Pharmacol..

[29] Kim E., Lee D.M., Seo M.J., Lee H.J., Choi K.S. (2021). Intracellular Ca^2+^ imbalance critically contributes to paraptosis. Front. Cell Dev. Biol..

[30] Liu N., Liu Y., Wang Y., Feng C., Piao M., Liu M. (2025). Oxidative cell death in the central nervous system: Mechanisms and therapeutic strategies. Front. Cell Dev. Biol..

[31] Wang L., Gundelach J.H., Bram R.J. (2017). Protein synthesis inhibition enhances paraptotic death induced by inhibition of cyclophilins in glioblastoma cells. Cancer Cell Microenviron..

[32] Vandenabeele P., Galluzzi L., Vanden Berghe T., Kroemer G. (2010). Molecular mechanisms of necroptosis: An ordered cellular explosion. Nat. Rev. Mol. Cell Biol..

[33] Kudelova J., Fleischmannova J., Adamova E., Matalova E. (2015). Pharmacological caspase inhibitors: Research towards therapeutic perspectives. J. Physiol. Pharmacol..

[34] Yoon M.J., Lee A.R., Jeong S.A., Kim Y.-S., Kim J.Y., Kwon Y.-J., Choi K.S. (2014). Release of Ca^2+^ from the endoplasmic reticulum and its subsequent influx into mitochondria trigger celastrol-induced paraptosis in cancer cells. Oncotarget.

[35] Moulder S. (2010). Intrinsic resistance to chemotherapy in breast cancer. Womens Health.

[36] Wang W., Fang H., Groom L., Cheng A., Zhang W., Liu J., Wang X., Li K., Han P., Zheng M. (2008). Superoxide Flashes in Single Mitochondria. Cell.

[37] Fontana F., Raimondi M., Marzagalli M., Di Domizio A., Limonta P. (2020). The Emerging Role of Paraptosis in Tumor Cell Biology: Perspectives for Cancer Prevention and Therapy with Natural Compounds. Biochim. Biophys. Acta (BBA) Rev. Cancer.

[38] Sperandio S., Poksay K., de Belle I., Lafuente M.J., Liu B., Nasir J., Bredesen D.E. (2004). Paraptosis: Mediation by MAP Kinases and Inhibition by AIP-1/Alix. Cell Death Differ..

[39] Fried L.E., Arbiser J.L. (2009). Honokiol, a Multifunctional Antiangiogenic and Antitumor Agent. Antioxid. Redox Signal.

[40] Wang C., Dai S., Zhao X., Zhang Y., Gong L., Fu K., Ma C., Peng C., Li Y. (2023). Celastrol as an Emerging Anticancer Agent: Current Status, Challenges and Therapeutic Strategies. Biomed. Pharmacother..

[41] Zhao H., Xu X., Lei S., Shao D., Jiang C., Shi J., Zhang Y., Liu L., Lei S., Sun H. (2019). Iturin A-like Lipopeptides from Bacillus Subtilis Trigger Apoptosis, Paraptosis, and Autophagy in Caco-2 Cells. J. Cell. Physiol..

[42] Wang Y., Wen X., Zhang N., Wang L., Hao D., Jiang X., He G. (2019). Small-Molecule Compounds Target Paraptosis to Improve Cancer Therapy. Biomed. Pharmacother..

[43] Zhivotovsky B., Orrenius S. (2011). Calcium and Cell Death Mechanisms: A Perspective from the Cell Death Community. Cell Calcium.

[44] Motiani R.K., Abdullaev I.F., Trebak M. (2010). A Novel Native Store-Operated Calcium Channel Encoded by Orai3: Selective Requirement of Orai3 versus Orai1 in Estrogen Receptor-Positive versus Estrogen Receptor-Negative Breast Cancer Cells. J. Biol. Chem..

[45] DeHaven W.I., Smyth J.T., Boyles R.R., Bird G.S., Putney J.W. (2008). Complex Actions of 2-Aminoethyldiphenyl Borate on Store-Operated Calcium Entry. J. Biol. Chem..

[46] Bootman M.D., Collins T.J., Mackenzie L., Roderick H.L., Berridge M.J., Peppiatt C.M. (2002). 2-Aminoethoxydiphenyl Borate (2-APB) Is a Reliable Blocker of Store-Operated Ca^2+^ Entry but an Inconsistent Inhibitor of InsP3-Induced Ca^2+^ Release. FASEB J..

[47] Cüce-Aydoğmuş E.M., İnhan-Garip G.A. (2024). Investigation of the Effects of Blocking Potassium Channels with 4-Aminopyridine on Paclitaxel Activity in Breast Cancer Cell Lines. Cancer Rep..

